# The Association between Electroencephalography with Auditory Steady-State Response and Postoperative Delirium

**DOI:** 10.3390/jpm13010035

**Published:** 2022-12-24

**Authors:** Naohiro Arai, Takahiro Miyazaki, Shinichiro Nakajima, Shun Okamoto, Sotaro Moriyama, Kanta Niinomi, Kousuke Takayama, Jungo Kato, Itta Nakamura, Yoji Hirano, Minoru Kitago, Yuko Kitagawa, Tatsuo Takahashi, Hideyuki Shimizu, Masaru Mimura, Yoshihiro Noda

**Affiliations:** 1 Department of Neuropsychiatry, Keio University School of Medicine, Tokyo 160-8582, Japan; 2 Department of Anesthesiology, Keio University School of Medicine, Tokyo 160-8582, Japan; 3 Department of Neuropsychiatry, Graduate School of Medical Sciences, Kyushu University, Fukuoka 812-8582, Japan; 4 Department of Psychiatry, Faculty of Medicine, University of Miyazaki, Miyazaki 889-1692, Japan; 5 Institute of Industrial Science, The University of Tokyo, Tokyo 153-8505, Japan; 6 Department of Surgery, Keio University School of Medicine, Tokyo 160-8582, Japan; 7 Department of Cardiovascular Surgery, Keio University School of Medicine, Tokyo 160-8582, Japan

**Keywords:** auditory-steady state response, EEG, gamma activity, phase-locking factor, postoperative delirium

## Abstract

Delirium is a disorder of consciousness and a risk factor for cognitive dysfunction and poor prognosis. We hypothesized that preoperative gamma activities would be linked to postoperative delirium. We enrolled 71 subjects for elective surgery and recorded auditory steady-state response (ASSR) by electroencephalography (EEG) before the surgery and examined postoperative delirium with DSM-5. The EEG data were analyzed for baseline power, and ASSR evoked power (EP) and phase-locking factor (PLF) within the gamma range. Postoperative delirium was found in 18 patients (delirium group) but not in 53 patients (non-delirium group). There were no significant differences in the 40-Hz EP or PLF between the two groups. The baseline gamma activity negatively correlated with the 40-Hz PLF in the non-delirium group (ρ = −0.444, *p* < 0.01). The correlation between baseline gamma activity and 40-Hz EP was not significant in either the delirium or non-delirium group. In all patients, both preoperative PLF and EP had no significant correlations with the Delirium Rating Scale Revised-98 and the Memorial Delirium Assessment Measure at the post-operation, respectively. The disruption of the neurophysiological relationship between baseline gamma activity before sound stimuli and the PLF of the 40-Hz ASSR may be one of the potential neurophysiological indicators associated with postoperative delirium.

## 1. Introduction

Gamma oscillatory neural activity is known to reflect the balance between excitation and inhibition (E/I balance) [1,2]. The E/I imbalance in the neural circuits is suggested to be involved in the pathophysiology of major psychiatric disorders [2]. The E/I imbalance can be assessed with the 40-Hz auditory steady-state response (ASSR) using electroencephalography (EEG) or magnetoencephalography measures [2,3]. It is also suggested that the association between baseline gamma activity and ASSR reflects the function of N-methyl-D-aspartate (NMDA) receptors on parvalbumin-expressing inhibitory interneurons [4]. The E/I balance has been reported as an etiology and biomarker for various psychiatric disorders [5], and they also may be potentially associated with delirium.

Delirium is a severe neuropsychiatric syndrome and a disorder of consciousness that presents with an acute confusional state and has been defined as an organic brain syndrome with a sudden onset that lasts a relatively short period of time [6,7]. Since the early 20th century, it has also been referred to as a form of symptomatic psychosis by Karl Bonhoeffer and is currently included in the Diagnostic and Statistical Manual of Mental Health 5 (DSM-5) and the ICD-10 Classification of Mental and Behavioural Disorders as a psychiatric disorder [8,9,10]. While the reported prevalence of delirium in inpatients varies among studies, a meta-analysis noted that the overall prevalence of delirium was 23% [11]. Furthermore, delirium is not just a transient complication but is linked to poor prognoses, such as cognitive decline, dementia, and death [12,13,14,15]. In addition, the cost of delirium is also a serious problem [16]. Thus, it is urgent to find a clinical measure to detect and prevent the onset of delirium. 

Risk factors for delirium are divided into predisposing factors and precipitating factors [17]. Predisposing factors are unmodifiable factors such as age, brain injury, and psychiatric disorders [7]. Precipitating factors include relatively modifiable factors such as infections, drugs, alcohol, and environmental changes [7]. Delirium is thought to be caused by combinations of these factors and is usually not caused by a single factor [18]. However, delirium generally presents with a similar set of symptoms regardless of causes, suggesting a common neural mechanism. 

E/I imbalance assessed with the 40-Hz ASSR has been reported to be attenuated in patients with schizophrenia [2,19]. ASSR is an important biomarker for the pathophysiology of schizophrenia and other neuropsychiatric disorders [20,21]. Phase locking factor (PLF) and evoked power (EP) are indicators of ASSR and have been implicated concerning the E/I imbalance [2]. In particular, the PLF has been reported to have a strong inverse correlation with NMDA channel occupancy in rat studies [22]. Gamma oscillations are thought to contribute to attention, perception, and memory [23,24], and impaired gamma oscillations are postulated as a neurophysiological endophenotype underlying schizophrenia and Alzheimer’s disease, reflecting the disrupted balance between E/I neuronal circuit activities [25,26].

EEG, besides being used as a tool to detect delirium, is used to study neural network activity underlying delirium. Abnormalities in functional connectivity of EEG have been reported in delirium, primarily increased connectivity in the theta-band and decreased connectivity in the alpha-band [27]. Disrupted neural connectivity was suggested to be linked to delirium as the extended slow-wave activity from frontal to posterior brain regions [28]. However, no electrophysiological studies have examined the change in the gamma oscillation. 

We hypothesized that the disruption of the gamma oscillations is involved in the development of delirium. To test this, we recruited inpatients admitted for elective surgery and measured the 40-Hz ASSR the day prior to the operation. We examined whether the ASSR metrics measured by EEG would be related to the emergence of postoperative delirium. The primary objective of the present study was to examine the relationship between delirium and the neurophysiological indices of ASSR (mean EP and PLF). In addition, we examined the associations between pre-stimulus EEG and the PLF in relation to postoperative delirium [4]. The secondary objective was to explore whether the ASSR-related indices could be related to the severity and cognitive declines in this pathological condition.

## 2. Materials and Methods

### 2.1. Participants

We designed a prospective cohort study on delirium in accordance with the Declaration of Helsinki and received approval from the Keio Faculty of Medicine Ethics Committee in December 2017. We recruited patients from December 2018 through March 2022. The inclusion criteria were as follows: (1) aged 20 years or older at the time of consent; (2) judged to be capable of obtaining written consent by the doctors; (3) scheduled or currently hospitalized in Keio University Hospital; (4) scheduled to undergo elective open chest or abdominal surgery (including thoracoscopic and laparoscopic surgery) at Keio University Hospital. On the other hand, we excluded the patients who had (1) disorders of consciousness (evaluated by Glasgow Coma Scale); (2) cerebral disease/injury being treated or in the past; (3) psychiatric disorders under treatment; (4) epilepsy being treated or history of epilepsy; (5) benzodiazepines, Z-drugs, antidepressants, and steroids administered within 14 days before surgery; (6) the score of the Japanese version of the Mini-Mental State Examination (MMSE) ≤23 or inability to comprehend the research as determined by the explainer [29,30]; (7) inability to understand Japanese language; (8) a history of vagus nerve reflex at blood collection; (9) inability to remain at rest during the EEG test; or (10) hearing loss. The patients taking benzodiazepines (for anxiolytics and/or hypnotics), Z-drugs, antidepressants, and steroids before the operation were excluded because the medications have been considered risk factors for delirium [31,32,33]. We obtained written informed consent from the included patients who met the eligible criteria. After the enrollment, EEG was measured one day before the operation on average. At the same time, we examined the neuropsychological tests as follows: MMSE, the Japanese version of the Delirium Rating Scale Revised-98 (DRS-R98) [34,35], and the Japanese version of Memorial Delirium Assessment Scale (MDAS) [36,37], Charlson comorbidity index [38], Barthel index [39] were also examined. We assessed for the presence of delirium on postoperative days 2–5 according to the DSM-5 based on at least one examination, information from nurses and other co-medicals, and medical record information each day [8]. When patients declined our visits, we had to hold off on these visits to minimize as much as possible the psychological and physical burden on them by our visits. This was because they had just undergone surgery. In such cases, we evaluated the patient at another time of the day or referred to information from the paramedical staff or medical records. When we determined that a patient had delirium, a psychiatric liaison team in our hospital also saw the patient for diagnosis. After that, patients were classified into the delirium and non-delirium groups based on all the evaluations. Patients were classified into the delirium and non-delirium groups based on the diagnosis. We used the DRS-R98 and MDAS to score the severity of delirium [34,36]. The Charlson Comorbidity Index was scored to assess the physical comorbidity history [38]. The Barthel index, the measure of disability, was obtained to evaluate the activities of daily living [39]. A higher score on the Charlson Comorbidity Index or a lower score on the Barthel index is known as a risk for postoperative delirium [40,41]. We also gathered clinico-demographic data (such as sex, age, body mass index (BMI), educational year, and surgical site) and clinical lab data (such as C-reactive protein, hemoglobin, and albumin) from the medical records. The information on smoking, alcohol use, and past history of delirium was based on the self-reports from the patients and their medical records. We classified patients who reported that they had not quit smoking at the point of admission as active smokers [42,43]. DRS-R98 and MDAS were performed as postoperative assessments at the moment of delirium assessment.

### 2.2. EEG Recordings

We recorded EEG data in 180 seconds using 19 active electrodes and the Polymate MP6100 EEG system (Miyuki Gi-ken, Japan) according to the standard 10–20 system placement. The patients were seated in the quiet room and instructed to be awake, relax, close their eyes, and receive passively auditory stimulation. We adapted a method used in a previous study to record the ASSR [35]. The auditory stimulations were composed of 40-Hz click sounds of 500 ms duration delivered 150 times binaurally through earphones at 80 dB sound pressure level using SuperLab ver. 5.0 (Cedrus Corporation, USA).

### 2.3. EEG Analysis

**Figure 1:**
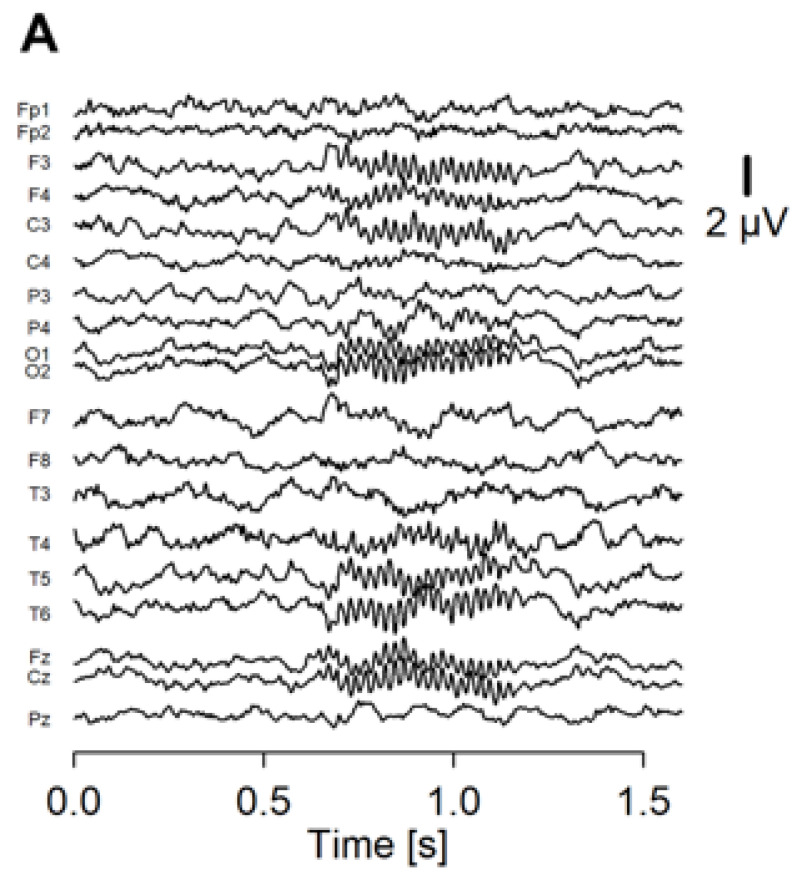

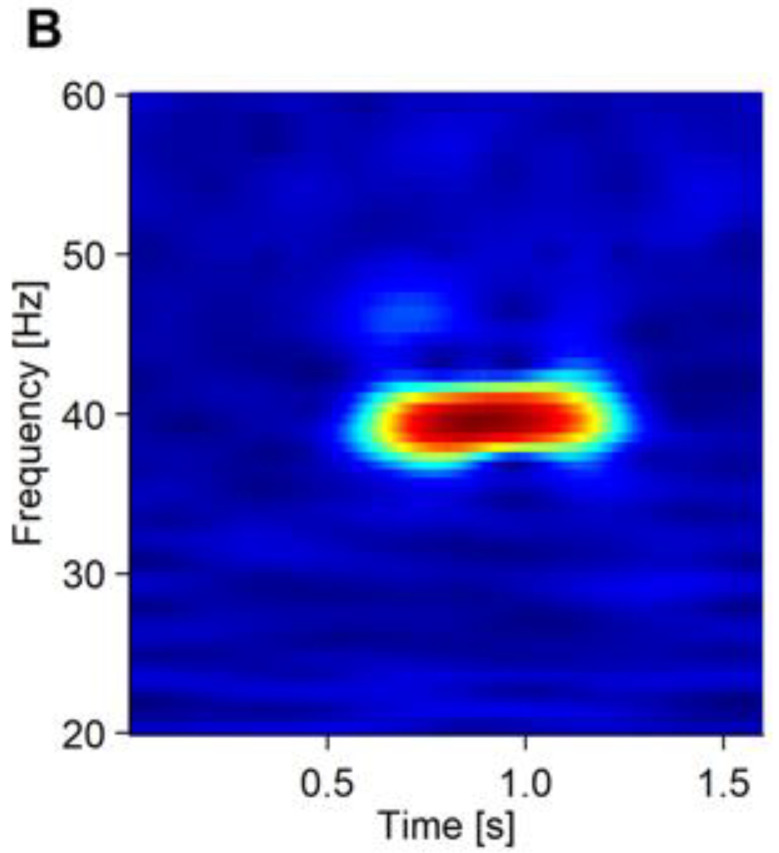
The averaged waveform and PLF of the ASSR of a representative patient. (**A**) Averaged waveform of the ASSR from 19 electrodes. The onset of the stimulus was at 0.5 ms of the *x* axis. (**B**) PLF of the ASSR shown in a heatmap.

Recorded EEG was analyzed using an in-house program run on R (https://www.r-project.org/ (accessed on 1 January 2020)). We separated the EEG data into 2-s epochs spanning −600 ms before to 1000 ms after the stimulus onset. Noisy epochs were automatically detected with the following criteria: (1) the peak-to-peak amplitude exceeds 200 μV from 1-Hz to 100-Hz frequency range; (2) the peak-to-peak amplitude exceeds 60 μV from 30-Hz to 50-Hz frequency range. The detected epochs were then visually checked, and up to 15 noisy epochs were rejected. Next, we performed independent component analysis on the accepted epochs. We removed up to 4 components that were thought to represent blink, eye-movement, and electromyographic artifacts. A wavelet transform was applied on each epoch to calculate EP (Figure 1A) and PLF (Figure 1B) as ASSR measures. 

As for the PLF, we can statistically analyze the phase locking of high-frequency components regardless of their amplitude and obtain the measurements of phase synchronization of EEG activity between trials at specific time intervals and frequencies [44,45]. Besides, we calculated the pre-stimulus EEG to acquire baseline amplitude. We applied the Hann window and Fast Fourier Transform on the pre-stimulus EEG data (−540 ms~−20 ms). The calculated data after the Fast Fourier Transform was averaged into baseline amplitude. Regarding the EP and PLF, we extracted and averaged the EP and PLF data for each patient at the Cz electrode in the time range from 100 ms to 600 ms after the stimulus onset and the frequency range from 38-Hz to 42-Hz. We compared the EP and PLF of the ASSR at the Cz between the delirium and non-delirium groups. Next, we also extracted and averaged the baseline gamma-band amplitude of each patient at the Cz electrode site with 30-Hz to 50-Hz as the frequency band of interest. We conducted correlation analyses to examine whether the pre-stimulus gamma-band activity can associate with the EP and PLF during the 40-Hz ASSR. 

### 2.4. Statistical Analysis

We used R programming language for statistical analysis (https://www.r-project.org/ (accessed on 1 January 2020)). In this study, all continuous variables were assessed with the Shapiro–Wilk test. The results showed that only the PLF data for the 40 Hz ASSR showed normality, while the other data showed nonparametric distributions. Continuous and categorical variables of the clinico-demographic data between the delirium and non-delirium groups were tested with Wilcoxon rank-sum tests and chi-square tests, respectively. EP and PLF in the 40-Hz ASSR were compared between the delirium and non-delirium groups using the Wilcoxon rank-sum tests and t-tests, respectively. We calculated Spearman correlation coefficients between the baseline gamma amplitudes and the mean 40-Hz ASSR results (EP and PLF) within each group because almost all the EEG data were nonparametric. For the subanalysis, we also calculated Spearman correlation coefficients between 40-Hz EP and PLF at the pre-operation and post-operative severity (DRS-R98 and MDAS scores) in the delirium group. We considered a two-tailed threshold of *p* < 0.05 as statistically significant. The continuous variables are described as mean scores and standard deviations (SD). We excluded the missing values from the statistical analysis.

## 3. Results

### 3.1. Clinico-Demographic Data

**Figure 2:**
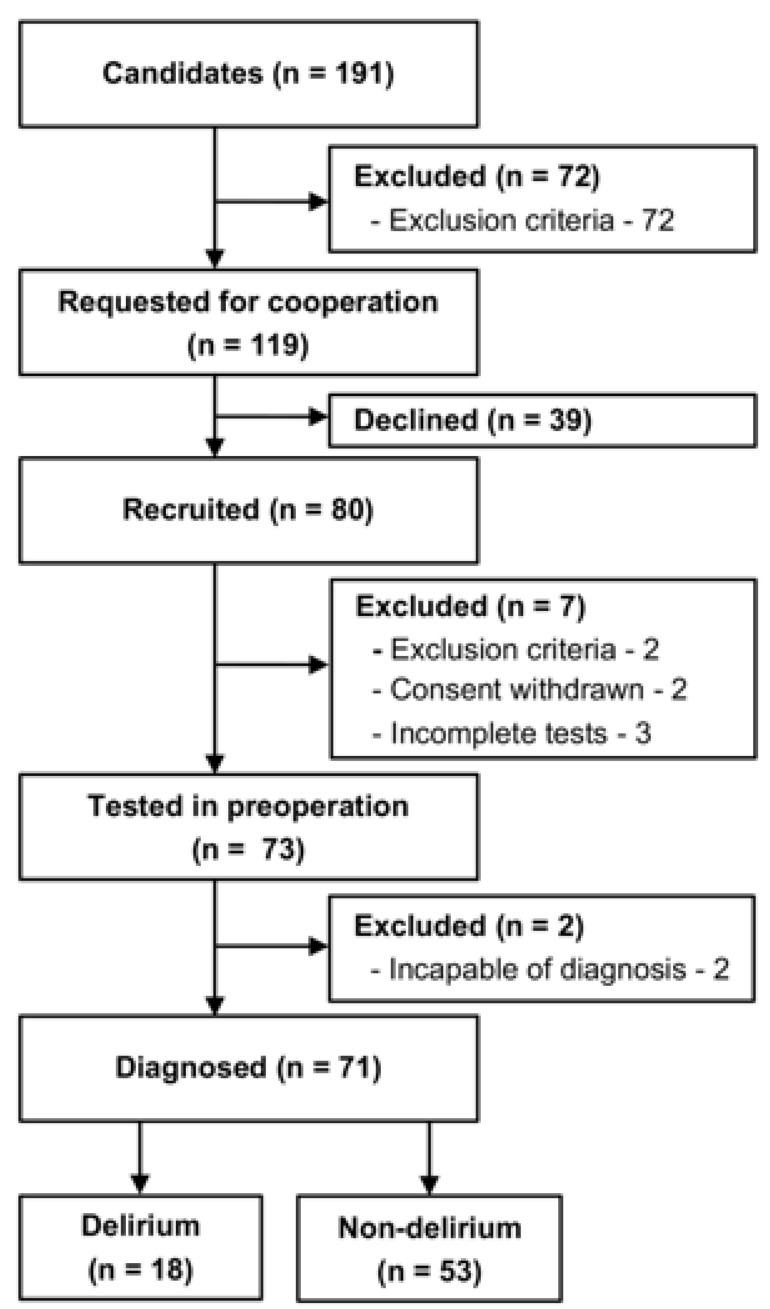
Recruitment flow chart. Eighty patients were recruited, and 73 patients were tested in the preoperative period. The emergence of delirium was assessed during postoperative days 2–5. Finally, 18 patients were classified into the delirium group, and 53 patients into the non-delirium group.

We recruited 80 patients before elective surgery (Figure 2). Nine (11.3%) patients were excluded because of exclusion criteria (MMSE below 23) (*n* = 2), withdrawal of consent (*n* = 2), incomplete tests (*n* = 3), or incapability of diagnosis for delirium (reoperation and postoperative complication) (*n* = 2). As a result, 71 patients (88.8%) were included in the analysis, of which 18 (25.4%) and 53 (74.6%) patients were included in the delirium and non-delirium groups, respectively. Delirium tremens was denied in all 71 patients. One of the 18 patients (5.6%) in the delirium group and 8 of the 53 patients (15.1%) in the non-delirium group declined the DRS-R98 examination after the operation. Three of the 18 patients in the delirium group (16.7%) and 12 of the 53 (22.6%) patients in the non-delirium group declined the MDAS examination after the operation. Demographic and clinical information is summarized in Table 1. The mean age was 76.6 ± 4.7 years in the delirium group and 72.3 ± 9.9 years in the non-delirium group. The delirium and non-delirium groups included 27.8 (%) and 30.2 (%) females, respectively. Compared with the non-delirium group, the delirium group had shorter years of education (delirium: 13.1 ± 3.1 years, non-delirium: 15.2 ± 2.5, *p* = 0.029), while no significant differences were found in the other comparisons. In comparing the characteristics of those who declined to score with those who did not in all patients, only the educational year was significantly lower in the former (DRS-R98: *p* < 0.01, MDAS: *p* = 0.047).

### 3.2. Neurophysiological Measures with ASSR

**Figure 3:**
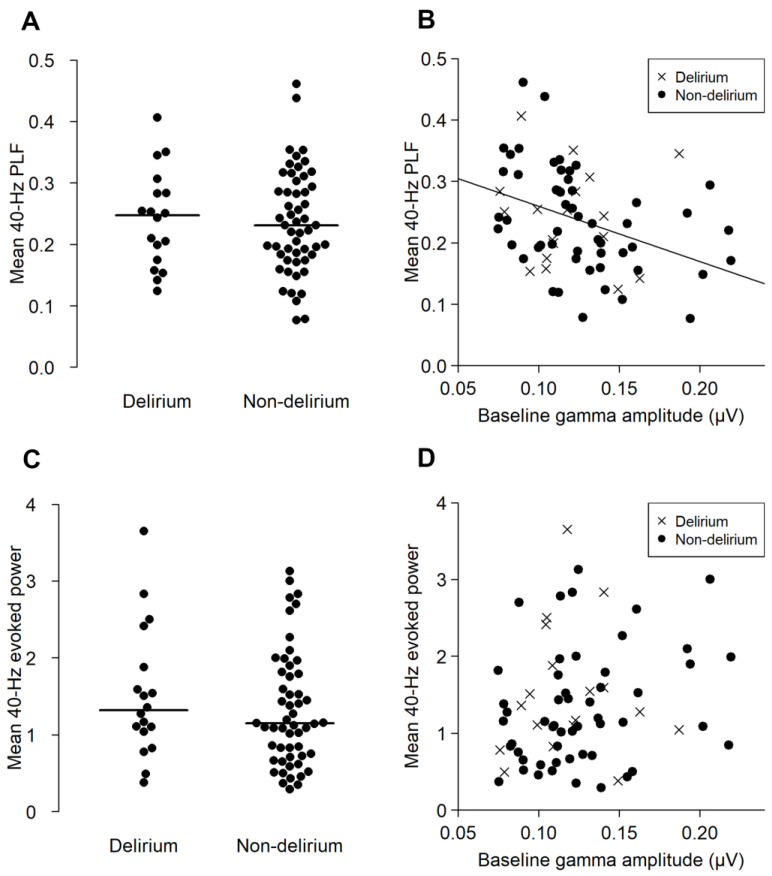
The PLF and evoked power (EP) of the ASSR of the delirium and non-delirium groups, and their correlation with the baseline gamma activity. The upper panels (**A**,**B**) represent the results of the mean 40-Hz PLF. The lower panels (**C**,**D**) represent the results of the mean 40-Hz EP. (**A**,**C**) show the distribution of the mean 40-Hz PLF (**A**) and mean 40-Hz EP (**C**) in the delirium and non-delirium groups. The horizontal lines indicate the median. There were no statistically significant group differences. (**B**,**D**) shows the correlation between the baseline gamma activity (*x* axis) and ASSR PLF ((**B**), *y* axis) and EP ((**D**), *y* axis). A significant correlation between the baseline gamma amplitude and PLF was found for the non-delirium group (ρ = −0.444, *p* < 0.001), but not in the delirium group (ρ = −0.120, *p* = 0.609).

There were no significant differences in 40-Hz PLF (t = 0.236, *p* = 0.815, 95% confidence interval (95% CI) −0.055–0.065) or 40-Hz EP (W = 547, *p* = 0.358, 95% CI −0.232–0.567) between the delirium and non-delirium groups (Figure 3A,C). The baseline gamma-band activity negatively correlated with the 40-Hz PLF in the non-delirium group (ρ = −0.444, *p* < 0.01), but not in the delirium group (ρ = −0.12, *p* = 0.609) (Figure 3B). On the other hand, the association between baseline gamma activity and 40-Hz EP was not significant in either the delirium (ρ = 0.073, *p* = 0.773) or non-delirium group (ρ = 0.255, *p* = 0.07) (Figure 3D).

### 3.3. Clinical Measures for Delirium

Clinical measures of the patients are summarized in Table 2. The DRS-R98 and MDAS scores after the operation were higher in the delirium group than in the non-delirium group (DRS-R98: W = 748, *p* < 0.001 95% CI 1.00–13.0, MDAS W = 584, *p* < 0.001, 95% CI 6.00–8.00) (Table 2). We performed a spearman’s correlation analysis of the postoperative DRS-R98 and MDAS scores for PLF and EP at the pre-operation in the delirium group, respectively. This analysis showed no significant correlations with DRS-R98 (*n* = 62, PLF: ρ = −0.025, *p* = 0.846, EP: ρ = 0.113, *p* = 0.386) and MDAS (*n* = 55, PLF: ρ = −0.043, *p* = 0.759, EP: ρ = 0.097, *p* = 0.485).

## 4. Discussion

The present study was a prospective observational study to examine whether EEG ASSR gamma activity in the preoperative period could relate to postoperative delirium. As a result, we observed a negative correlation between prestimulus baseline EEG gamma activity and the PLF of the 40-Hz ASSR in the preoperative period in the non-delirium group, whereas the relationship was not statistically significant in the delirium group. This may suggest that the relationship between gamma power and synchronization to auditory stimuli may have been disrupted even preoperatively. This may suggest that the relationship between gamma power and synchronization to auditory stimuli may have been disrupted even preoperatively, and gamma-band activities in patients at risk of delirium can be relatively restricted to the cortex involved in sensory perception. This disruption might be a biomarker to predict postoperative delirium.

The strength of this study was the strict eligibility criteria for delirium. Since the pathophysiological conditions of delirium are heterogeneous, it is crucial to conduct research with some controlled eligible criteria. To date, few previous studies analyzed preoperative EEG in relation to postoperative delirium. One study demonstrated a decreased frontal alpha power and alpha band connectivity in patients with postoperative delirium [28]. There has been no research reported about event-related EEG including ASSR. This is the first clinical study to evaluate the risk of postoperative delirium in terms of preoperative EEG ASSR measures [27,46].

Previous studies showed that pre-stimulus baseline EEG is related to the ASSR [4,19]. The present study demonstrated that, in the non-delirium group, there was a negative correlation between baseline gamma-band activity prior to auditory stimulation and ASSR. In contrast, the delirium group showed no significant relationship between pre-stimulus gamma power and the EP or PLF in the 40-Hz ASSR. These results might indicate that gamma-band oscillation and its response to auditory stimuli are altered among the patients who are at risk of delirium. It seems natural to assume that a larger baseline gamma activity would result in a larger 40-Hz ASSR. In fact, the evoked ASSR amplitude tended to be larger for patients with a larger baseline gamma activity, although this effect was not statistically significant. The discrepancy between the evoked ASSR and PLF in their relationship with the baseline gamma activity may be explained by assuming multiple sources of baseline gamma activities. A larger baseline gamma amplitude may have reflected an increase in gamma activities that is unrelated rather than related to auditory perception. Such activity may have blurred the auditory response in the gamma range, thus resulting in more inconsistent phases of gamma activity across the trains of stimuli and a smaller PLF. In contrast, evoked ASSR should reflect the size of synchronized activity, not the ratio of the auditory gamma activity to the total gamma activity. However, the negative correlation between the baseline gamma activity and ASSR was not observed in the delirium group. This may indicate the gamma-band activities in patients at risk of delirium are relatively restricted to those involved in sensory perception. In such patients, inhibitory tone might have attenuated higher-order neural processing reflected in gamma-band activities. Gamma oscillatory neural activity is known to reflect inhibitory tone [1,2]. Inhibitory tone in the neural circuits is regulated through GABAergic inhibitory interneurons to balance the glutamatergic excitatory neurons [47,48,49]. Inhibitory tone has also been proposed as a hypothesis of delirium by Sanders et al. [50]. In fact, benzodiazepines, which directly act on GABA mediated circuits, are a risk factor for delirium [51] and delirium is related to narrowed attention [7]. The present finding seems to be in line with past studies on gamma band activity in various psychiatric disorders [3,52,53]. 

There was no difference in the mean 40-Hz PLF between the delirium and non-delirium groups. This result suggests that gamma oscillatory activity may not be particularly reduced before the onset of delirium. Most of the previous ASSR studies have focused, specifically on schizophrenia. A meta-analysis of ASSR studies in schizophrenia showed that the 40-Hz ASSR tended to be lower in younger patients than in older patients [2]. The mean age of both groups in the present study (delirium: 76.6 years, non-delirium: 72.3 years) was higher than in the schizophrenia studies, suggesting that the effects of aging on the ASSR measures in our sample could not be ruled out [2]. Even if there were no significant group differences in preoperative gamma oscillatory findings between the delirium and non-delirium groups, it remains unclear whether delirium is neurophysiologically distinct from other psychiatric disorders, such as schizophrenia or bipolar disorder [3]. Given the focus of this study on the prediction of delirium, it may be helpful to compare studies that have examined the preclinical states of psychiatric disorders with the ASSR. At present, no study investigated the preclinical state other than those at risk for developing psychosis [5]. These studies have reported the attenuated 40-Hz ASSR in this group, suggesting that 40-Hz ASSR may predict the development of psychosis, including schizophrenia and bipolar disorder [5,54,55,56]. The patients in this study were assumed to be those without a diagnosis of psychiatric disorder, and none of them exhibited hallucinatory or delusional symptoms, at least before surgery. It is possible that the 40-Hz ASSR was in the normal range because the patients in both groups in the present study might have different characteristics from those at risk for developing psychosis. On the other hand, one study reported no difference in gamma ASSR between those at clinical high risk for psychosis and healthy controls [5,57]. These may suggest that changes in neural activity, especially E/I imbalance, due to the prodromal phase may not always be reflected in the simple 40-Hz ASSR. Instead of a simple analysis of the 40-Hz ASSR, a more detailed analysis of the ASSR, such as the combination with baseline gamma activity in this study, the phase-amplitude coupling, and the functional connectivity, may find predictive potential. It may be helpful to compare delirium with organic, including symptomatic, mental disorders that are not defined as psychiatric disorders but can lead to delirium, such as encephalitis and neuropsychiatric systemic lupus erythematosus, [10,58,59,60] but no studies have so far measured ASSR in humans. One study reported that when antibodies from a patient with central lupus were administered to mice with reduced microglial activity, the 40 Hz ASSR of those mice was reduced [61]. Another study in which mice were treated with antibodies from human LGI1 limbic encephalitis suggested that LGI1 may regulate the balance between excitation and inhibition [62]. It was suggested that a decrease in the activity of LGI1 might reduce the activity of the inhibitory network rather than the excitatory network [62]. From these, the 40-Hz ASSR in symptomatic psychosis may decrease due to decreased activity of the inhibitory network as a result of decreased microglial activity. Since delirium has also been suggested to be related to changes in microglial activity [7], a reduction in ASSR may be seen when measured during delirium. However, this was not done in this study.

The current study found no significant differences in most clinical backgrounds between the two groups, while we excluded those with preoperative cognitive impairment by the eligibility criteria. However, the only item, years of education, was smaller in the delirium group. Educational attainment is not considered a main predisposing factor. Still, a retrospective cohort study by Jones et al. reported each year of completed education was associated with 0.91 lower odds of delirium [63]. In addition, a review study by Feinkohl et al. suggests that long years of education may decrease the risk for postoperative cognitive dysfunction [64]. These reports implicate that short years of education could be one of the predisposing factors for delirium. Postoperative severity was significantly higher in the delirium group than in the non-delirium group. However, the severity of the delirium group in the present study may have been relatively mild. The cutoff of the Japanese version of the DRS-R98 was reported 14.5, whereas the mean in this study was 15.6 [35]. The Japanese version of the MDAS has a cutoff of 10, whereas the mean in this study was 9.6 under the cutoff [37]. This was thought to be because the diagnosis in this study was based on the DSM-5 and was not related to severity, as delirium was judged to be delirium when the diagnostic criteria were met. However, it is important to consider the potential for subsyndromal delirium. Subsyndromal delirium is defined as the presence of one or more symptoms of delirium that do not meet the criteria for a full diagnosis of delirium [65]. It is possible that patients with subsyndromal delirium were overdiagnosed as part of the delirium group due to our limited observation and diagnostic abilities, leading to a lower mean severity score for the delirium group than expected. It is also possible that the level of surgical invasion may be lower and perioperative care may have advanced since the time these severity score validity assessments were made. No significant correlations were found between preoperative PLF and EP and these postoperative severities. Almost all the ASSR studies for psychiatric disorders have employed the cross-sectional design. As such, this was the first prospective study for predicting neuropsychiatric conditions with the ASSR, warranting further research.

There are several limitations in the current study. First, the sample size of this study was small. This is because we set strict inclusion and exclusion criteria to ensure the quality of our delirium study. Based on the preliminary nature of this pilot study, a larger sample size would have been required. A study with a larger sample size in the future is needed to lead to the development of ASSR indices (effect size: EP = 0.11, PLF = 0.03) that can contribute to the prediction of delirium. Second, since this was an observational study in the clinical setting, there was a limitation in that the details of the operation and the patients’ backgrounds could not be controlled entirely. Third, although the inclusion in this study was over 20 years of age, the age of the patients included in the study was higher as a result. The patients who were scheduled to undergo surgery at this hospital were predominantly older; moreover, there was only one young patient (30 s) who agreed to be asked to participate in this study. Therefore, sampling bias is present in this study. Future improvements include reanalysis of the patients restricted to those over 65 or 70 years of age. Fourth, the occurrence of delirium in this study was lower than expected compared to the general incidence of delirium a decade ago, making it difficult to enroll a sufficient number of patients with delirium. This may be due to the fact that surgical techniques have become less invasive than in the past and that many surgical wards have taken preventive measures against the onset of delirium. The evaluation date could also be a possible cause of this limitation. Although it is commonly understood that postoperative delirium occurs on days 2–5 [66], it is possible that delirium appeared as early as day 1. Therefore, we could not rule out the possibility that the number of delirium groups was reduced. In addition, because the patients were postoperative, we could not evaluate them at the same time every day for an extended period of time, taking into consideration their wishes and the psychological and physical burden on them. Therefore, the limitations of this study were that it was based solely on the evaluation of the presence or absence of delirium based on intermittent observations and that we could not monitor the patients consistently and continuously postoperatively. Fifth, although the present study excluded patients taking any of the medications listed as a predisposing factor, most patients took numerous other medications, such as antihypertensive and antihyperlipidemic, which may still have been a confounding factor for delirium. Sixth, we could not adequately examine the changes in cognitive function before and after surgery. In this study, MMSE could not be assessed postoperatively from all patients in both the delirium and non-delirium groups, preventing us from examining differences in cognitive function between preoperative and postoperative periods. In addition, the DRS-R98 and MDAS scores were evaluated only 83.3% and 94.4%, respectively, of the time in the delirium group because of their decline. Those who had the MDAS examination were significantly older than those who did not. The significant educational year difference may have affected our results. Seventh, the subtypes of delirium were not considered in this study. Delirium is classified into three subtypes according to psychomotor symptoms: hyperactive, hypoactive, and mixed types [6,8], which may be associated with different pathophysiologies [67]. Eighth, a potential problem in ASSR measurement is the limitation that the measurement environment and patient conditions can easily contaminate electromyograms and other noise. In addition, EEG is characterized by high temporal resolution but relatively low spatial resolution. In this study, we used 19 electrodes using the international 10–20 method, which has certain limitations regarding spatial information. However, since the ASSR in this study set the Cz electrode site as the region-of-interest, this problem is not a particularly significant limitation. In previous ASSR studies, ASSR metrics have often been measured at the Fz, Cz, or FCz electrode sites in the International 10–20 method [54], as these are the electrode sites that can stably measure ASSR findings. Furthermore, the Cz electrode site has the advantage of being the least susceptible to electromyographic noise. Furthermore, potential hearing impairments that the patients may have could affect the ASSR results. The patients with hearing impairment were excluded from the beginning of this study. However, the effect of age-related hearing loss cannot be ruled out, even if none of the patients had an obvious hearing impairment. This was because many of the patients included in this study were elderly. Such possibility should be eliminated by rigorous audiometric examinations as a future measure. Ninth, medications may have been a confounding factor. We excluded the drugs with anticholinergic effects nor GABAergic sedative effects at the recruitment [7]. In addition, no patient in this study was taking acetylcholine inhibitors or antipsychotics (sometimes used for preventing delirium). However, other medications were not completely controlled. As this study was designed for patients undergoing surgery, the influence of drugs used in the perioperative period, including the intensive care unit, on the occurrence of delirium could not be ruled out. Medications for the treatment or control of their primary diseases, such as hypertension, hyperlipidemia, and diabetes, also could not be excluded. This may have caused or prevented the development of delirium.

## 5. Conclusions

In summary, we found that the relationship between baseline gamma activity and PLF in the 40-Hz ASSR could be one of the surrogate indicators surrogate indicator for postoperative delirium. However, in the future, it is necessary to improve the study design to be more rational such as by aligning the numbers of both groups as much as possible before analyzing the data. Further studies employing the ASSR modality may help identify biomarkers contributing to the early detection of postoperative delirium and elucidate its mechanisms.

## Figures and Tables

**Table 1:** Clinico-demographic data.

	Delirium (*n* = 18)	Non-Delirium (*n* = 53)	*p*
Sex, Female (%)	5 (27.8)	16 (30.2)	1.000
Age (mean ± SD, years)	76.6 ± 4.7	72.3 ± 9.9	0.154
Body Mass Index (mean ± SD)	22.0 ± 2.6	23.3 ± 3.2	0.467
Educational year (mean ± SD, years)	13.1 ± 3.1	15.2 ± 2.5	0.029
Surgical site, Thoracic surgery (%)	17 (94.4)	42 (79.2)	0.262
Past history of delirium (%)	4 (22.2)	2 (3.78)	0.052
Active smoker (%)	3 (16.7)	3 (5.6)	0.337
Alcohol in 2 weeks (%)	9 (50)	27 (51)	1.000
Hemoglobin (mean ± SD, mg/dL) ^a^	12.0 ± 2.6	12.9 ± 1.2	0.101
C-reactive protein (mean ± SD, mg/dL) ^a^	0.29 ± 0.38	0.23 ± 0.49	0.079
Albumin (mean ± SD, g/dL) ^a^	3.8 ± 0.4	4.0 ± 0.4	0.085

Notes. Continuous and categorical variables of the clinico-demographic data between the delirium and non-delirium groups were tested with Wilcoxon rank-sum tests and chi-square tests, respectively. Significant differences were not observed except for the item of the educational year (*p* = 0.029). ^a^: Blood test results before the operation.

**Table 2:** Pre- and post-operative clinical measures of the patients.

Delirium	Delirium	Non-Delirium	*p*
Pre MMSE	27.8 ± 1.6	28.2 ± 1.5	0.373
Charlson Comorbidity Index	1.3 ± 1.6	1.2 ± 1.4	0.961
Barthel index	99.4 ± 2.4	100 ± 0.0	0.092
Post DRS-R98	15.6 ± 2.76	4.2 ± 2.82	<0.01
Post MDAS	9.6 ± 1.26	2.6 ± 1.79	<0.01

Notes. Continuous variables of the clinical measures (mean ± SD) between the delirium and non-delirium groups were tested with Wilcoxon rank-sum tests. Significant differences were not observed except for the item of the DRS-R98 and MDAS in the postoperative period (*p* < 0.01). Abbreviations: DRS-R98, the Japanese version of the Delirium Rating Scale Revised-98; MDAS, the Japanese version of Memorial Delirium Assessment Scale, the Japanese version of the Mini-Mental State Examination; Post, postoperation; Pre, preoperation.

## Data Availability

Clinical and EEG data in this study are available upon reasonable request to the corresponding author.
